# Bench to Bedside: Proteomic Biomarker Analysis of Cerebrospinal Fluid in Patients With Spondylomyelopathy

**DOI:** 10.7759/cureus.16003

**Published:** 2021-06-28

**Authors:** Brian Fiani, Claudia Covarrubias, Ryan Jarrah

**Affiliations:** 1 Neurosurgery, Desert Regional Medical Center, Palm Springs, USA; 2 School of Medicine, Universidad Anáhuac Querétaro, Santiago de Querétaro, MEX; 3 Neurological Surgery, University of Michigan - Flint, Flint, USA

**Keywords:** vitamin d-binding protein, gelsolin, creatine kinase b-type, angiotensinogen, pigment epithelium-derived factor, prostaglandin-h2 d-isomerase, apolipoprotein e, clusterin, spondylomyelopathy, proteomics

## Abstract

Establishing proteomic biomarkers is critical for characterizing disease pathophysiology, identifying genetic risk factors, and predicting clinical outcomes. However, diseases like cervical spondylomyelopathy have not been actively characterized for molecular significance, leading to questions regarding the pathology’s molecular mechanisms. Namely, spondylomyelopathy is a degenerative spinal disease that leads to compression and neurologic deficits in the spinal cord. Analyzing a patient’s cerebrospinal fluid (CSF) has been well-known for revealing biomarkers that are associated with diseases of the central nervous system. Therefore, in this review, we will formulate a proteomic profile of spondylomyelopathy through a molecular analysis of the CSF. The proteins found to be upregulated in the CSF include vitamin D-binding protein (VDBP), gelsolin, creatine kinase B-type (CK-BB), and angiotensinogen. Meanwhile, the proteins that were downregulated include pigment epithelium-derived factor (PEDF), prostaglandin-H2 D-isomerase (PGH2), apolipoprotein E (APOE), and clusterin. The cellular functions of these proteins are discussed, along with their relevance in manifesting spondylomyelopathy. However, further studies are warranted, as a lack of human studies is a major limiting factor. Nevertheless, based on the continued progression of the proteomic profile of spondylomyelopathy, new targets can be assessed as candidates for future therapeutic intervention.

## Introduction and background

Cervical spondylotic myelopathy (CSM) is defined as a neurological degenerative condition characterized by spinal canal narrowing and progressive spinal cord compression, resulting in clinical myelopathy with or without associated radiculopathies [1,2]. CSM can occur in two forms: disc-associated CSM and osseous-associated CSM (OA-CSM). The clinical signs can present as acute or slow-progressing with variable neck pain, ataxia, and, in some cases, paresis or paralysis of all limbs [3]. Given that there is currently limited knowledge highlighting the molecular mechanisms underlying this spinal cord condition, identifying novel biomarkers could become a more accessible diagnostic modality.

Proteomics, defined as the systematic and large-scale analysis of proteins and their multitude of properties, including expression, activity, modification, and interactive properties, is a gateway that is functionalizing the genome [4]. In order to further understand diseases, it is fundamental to determine the protein activity and specific molecular mechanisms underlying the disease phenotypes [5]. Unlike transcriptomics and genomics, proteomics is superior as it is capable of identifying post-translational modifications, leading to its utility in identifying causative factors in disease pathogenesis and biomarkers alike [6]. The proteins are typically extracted from the biological samples of interest, such as cells, tissues, and bodily fluids, and undergo peptide separation and processing through sophisticated software, yielding protein identification [5]. With the use of mass spectrometry and chromatographic techniques, approaches in proteomics that utilize cerebrospinal fluid (CSF) to further investigate the pathogenesis of neurological diseases, such as spondylomyelopathy, are rapidly evolving [6]. Furthermore, CSF protein content alterations can reflect tissue damage which can be useful in predicting the progression of neuroinflammatory diseases.

Comparative proteomics research is an emerging tool helping to identify proteome catalogs that will serve as references in the absence and presence of certain pathological processes [7]. Moreover, large animal models, such as canines, are physiologically more adept, when compared to rodent models, at producing multiple samplings available in sufficient quantities [3]. With the use of comparative proteomic veterinarian studies on canine models, recent prospective studies have successfully identified the potential protein expression in CSF samples obtained from CSM-affected dogs [3,8]. Such studies have identified both decreased and increased protein expression when comparing CSF samples of control dogs and CSM-affected dogs. Albeit currently limited, and considering that canine and human DNA are comparable, the research identifying specific protein expression through canine CSM studies can further bridge the gap between understanding the pathological mechanisms associated with the expression of CSM in humans [3]. Other diseases of the spinal cord can be confounding in the clinical diagnosis of CSM. Thus, the identification of CSM-specific biomarkers is imperative in order to facilitate the appropriate diagnosis and management.

When properly regulated, the proteins associated with CSM are integral to the normal pathophysiological mechanisms that drive cellular homeostasis, bone mineralization, and neuroprotection. Moreover, each of these proteins is involved with key molecular pathways that drive key cellular functions, such as DNA repair, apoptosis, vitamin transport, or energy production. Herein, the typical molecular profile of proteins associated with CSM is characterized. 

## Review

CSF protein expression associated with spondylomyelopathy

Recent studies have been able to identify proteins with differential expression from CSF samples taken from CSM-affected dogs [1,8]. When comparing CSF samples in both the control and CSM-affected groups, Martin-Vaquero et al. identified an increased expression of eight proteins, including angiotensinogen, vitamin D-binding protein, and gelsolin, and a decreased expression of four proteins, including apolipoprotein E and prostaglandin-H2 D-isomerase [1]. A list of the aforementioned proteins is summarized in Table 1 below. The results of this study can further indicate the relevance to the human counterpart of this condition and raise the relevance to the utility of proteomics and biomarkers. Subsequently, in 2019, Vansteenkiste et al. published a first-of-its-kind study characterizing the expression levels of microRNAs (miRNAs) present in CSF of canine models and their potential role in the pathogenesis of OA-CSM. Although results are limited, the authors were able to introduce a characterization of the miRNA profile in the CSF of the clinically normal dogs compared to the OA-CSM affected group [8].

**Table 1:** CSF proteome of CSM-affected dogs compared to clinically normal dogs. SPARC: secreted protein acidic and rich in cysteine; HS: Heremans-Schmid; CSF: cerebrospinal fluid; CSM: cervical spondylotic myelopathy

Increased Expression	Decreased Expression
Vitamin D-binding protein	Pigment epithelium-derived factor
Gelsolin	Prostaglandin-H2 D-isomerase
Creatine kinase B-type	Apolipoprotein E
Angiotensinogen	Clusterin
Alpha-2-HS-glycoprotein	
Glycoprotein SPARC	
Calsyntenin-1	
Complement C3	

Another possible protein of interest is the recombinant bone morphogenic protein-2 (rhBMP-2) that promotes bone creation and remodeling, increasing the likelihood of bony fusion without the need for autologous grafting [9]. Other forms of myelopathy, such as autoimmune myelopathy, have been attributed to the presence of several serological markers [10]. The collapsing response-mediator protein-5 immunoglobulin G (CRMP-5) has been suggested as a potential biomarker through both serological and CSF evaluations in patients presenting progressive or acute myelopathy [10]. Both of these proteins can be further evaluated in well-designed studies and help to breach the gap between finding disease-specific biomarkers for CSM.

Vitamin D-binding protein and gelsolin

Vitamin D-binding protein (VDBP) is the binding protein for vitamin D metabolites [11]. VDBP is a multifunctional protein that is well-conserved during the evolution of vertebrates [11]. VDBP works in tandem with vitamin D metabolites to promote the proper development and maintenance of bone tissue [12]. Deficiency in vitamin D is a key contributor to the disruptions in cellular homeostasis, leading to the clinical manifestations of hyperparathyroidism and chronic kidney disease [13]. Vitamin D is obtained through sources such as diet and sunlight; however, the metabolite is not biologically active until processed through the kidneys. The final result of 1,25-dihydroxy vitamin D3 is chemically unstable in the physiological environment and requires the activity of VDBP for transport to the target tissue [14]. Therefore, VDBP is crucial to the half-life and biological activity of the active vitamin D steroid molecule [15]. Once at the target tissue, vitamin D can be released from VDBP into the target cells, where it can be metabolized or form a complex with specific receptors. This complex can then transport to the nucleus and interact with transcription factors to influence gene expression or cellular activity [16].

Gelsolin is a member of the actin-binding protein family and is involved in maintaining and supporting cellular architecture and cell motility [17]. Gelsolin acts upon actin filaments by nucleating and capping their ends and is regulated by calcium and polyphosphoinositide ions [18]. Gelsolin has anti-apoptotic behavior by stabilizing mitochondria and preventing the release of cytochrome C, thereby inhibiting apoptosis [18]. Physiologically, gelsolin is not necessary for survival; however, it is essential for rapid responses of dynamic cells during wound healing and platelet formation. This means its function is critical for normal physiologic and cellular function [19].

**Figure 1:**
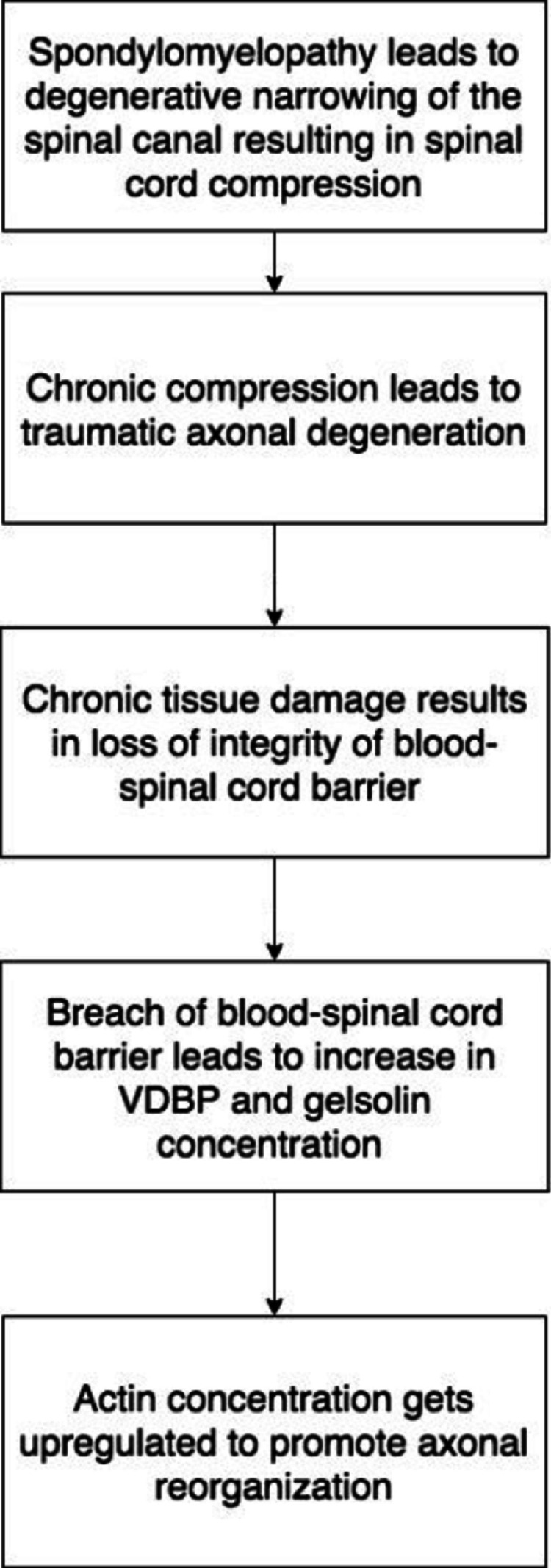
Mechanism of how the spondylomyelopathy leads to an increase in vitamin D-binding protein and gelsolin in the CSF of affected patients. CSF: cerebrospinal fluid; VDBP: vitamin D-binding protein

The role of VDBP and gelsolin in spondylomyelopathy follows an interesting mechanism that leads to its upregulation [1]. According to Martin-Vaquero et al., canine models were found to have upregulated VDBP in the CSF, indicating a breach in the blood-spinal cord (SC) barrier (Figure 1). In terms of function, gelsolin and VDBP are similar in the sense that they can regulate actin proteins. However, when axonal degeneration occurs, actin filaments are released, thereby increasing the expression of VDBP and gelsolin [1]. Therefore, when axonal damage occurs because of spondylomyelopathy, actin concentration increases in order to promote actin regeneration. This makes VDBP and gelsolin valuable proteins for CSF analysis.

Creatine kinase B-type and angiotensinogen

Creatine kinase B-type (CK-BB) and angiotensinogen are two other proteins that have increased expression with the manifestation of spondylomyelopathy [1]. Creatine kinase is a high-energy phosphorylating molecule that catalyzes the production of a phosphocreatine molecule [20]. Phosphocreatine is a high-energy reservoir that allows the supply of adenosine triphosphate (ATP) to biological systems of the heart, brain, skeleton, and muscles [21]. Creatine kinase B-type is an isoform of the enzyme creatine kinase that exists primarily in the brain [22]. When creatine kinase B dimerizes, it gains another polypeptide chain to form the CK-BB complex. CK-BB is associated with proper ion transport to the brain and is pivotal for energy homeostasis in brain tissue [23]. Elevated levels of CK-BB are an indicator of central nervous system diseases, and therefore it is considered a valuable biomarker.

Conversely, angiotensinogen (AGT) is among the proteins critical in the renin-angiotensin pathway and is produced by the liver [24]. On its own, the protein is a zymogen, requiring another enzyme to activate its enzymatic activity. AGT is the inactive precursor to angiotensin, a key peptide hormone that regulates blood pressure [24]. AGT is secreted in response to low blood pressure or adverse changes in sodium concentration [25]. Subsequently, renin is also secreted by the kidneys and activates AGT to form angiotensin I [25]. Angiotensin I is inactive and is further processed into the biologically active form of angiotensin II, which is subsequently used to allow the vasoconstriction of blood vessels [25]. Moreover, the binding of angiotensin II leads to aldosterone release, which further leads to sodium retention and an increase in blood pressure.

In reference to spondylomyelopathy, CK-BB has been found in patients with CSM, where elevated CK-BB levels being found within the CSF of affected canines [1]. Elevated CK-BB is associated with myelin degeneration and damage in white matter tissue, indicating that it could promote neural damage in the CNS [1]. Meanwhile, AGT is a serine protease inhibitor, which promotes cartilage destruction in patients with osteoarthritis [1]. This cartilage damage could evidently contribute to the degenerative changes that occur to the spinal canal during CSM’s pathogenesis.

Glycoprotein SPARC and complement C3

Glycoprotein secreted protein acidic and rich in cysteine (SPARC) is a multicellular glycoprotein that is involved with many biological processes [26]. Its main function is counteradhesion and anti-proliferation of endothelial, smooth muscle, mesangial, and fibroblastic cells [27]. Its counter adhesive function is achieved by the loss of the focal adhesion complex and is pivotal for cell survival by regulating cell shape [27,28]. SPARC has also been characterized to have a role in cell development, tissue remodeling, tissue repair, and cell turnover [28]. In addition, SPARC has a high affinity for calcium-binding proteins, leading to its conformational changes that reduce the affinity of the extracellular domain to proteinases, thereby altering the susceptibility to collagen proteins [29]. SPARC has also been studied to bind well to cytokines such as platelet-derived growth factor (PDGF) and vascular endothelial growth factor (VEGF) [28]. SPARC helps to regulate the biological functions of these growth factors, along with that of many other key proteins involved in cellular homeostasis [28].

Complement C3 is highly involved in the complement cascade system, the immunological pathway that is critical to attacking pathogens and promoting an inflammatory response [29]. Complement C3 is a zymogenic protein that is encoded by the C3 gene on chromosome 19 [30]. It is critical in activating the complement cascade for both the classical and alternative pathways, making its presence pivotal in fighting bacterial infection [29]. Complement C3 deficiency often leads to infection and weakens the innate immunity in patients [29].

Elevated SPARC levels have been seen to contribute to degenerative pathologies [1]. CSF analysis reveals that canine models with spondylomyelopathy have elevated SPARC, leading to vertebral changes that may disrupt the normal physiology of the cell [1]. In treated canines, elevated SPARC was not seen, indicating that SPARC can serve as a valuable biomarker in identifying spondylomyelopathy [1]. Complement C3 is also seen to be elevated in causing degenerative changes leading to myelopathies [1]. Overregulation of C3 has contributed to the host cell damage and accumulation of immunological debris [1]. This may lead to the development of nerve damage in the spinal canal. Inhibition of C3 has been studied to alleviate signs of arthritis in rodent models, with complement inhibition being a potential therapeutic model for degenerative spinal pathologies [1].

Pigment epithelium-derived factor and prostaglandin-H2 D-isomerase

Pigment epithelium-derived factor (PEDF) is a multifunctional protein that has been expressed in various tissues, such as the eye, bone, liver, heart, and adipose tissue [31]. PEDF is known to have neurotrophic and anti-angiogenic properties [31]. This means that PEDF promotes the development of neurons and inhibits the growth of blood vessels. PEDF is also well characterized to be involved in neuroprotection, fibrogenesis, inflammation, angiogenesis, and organogenesis [31]. PEDF is highly expressed in bone marrow, making it function in bone remodeling and bone mineralization [31].

Meanwhile, prostaglandin-H2 D-isomerase (PGH2) is a key enzyme involved in the maintenance of the central nervous system [32]. PGH2 is used to convert prostaglandin H2 to prostaglandin D2. The function of prostaglandin D2 is to modulate the activity of the CNS, regulate platelet aggregation, and apoptosis, along with supporting muscle contraction and relaxation [32].

As indicated in the study by Martin-Vaquero et al., the relevance of PEDF and PGH2 to spondylomyelopathy is that their expression is decreased in CSF analysis [1]. PEDF is known to be a neuroprotector for spinal cord neurons from cellular-induced injuries [1]. This makes PEDF a target protein for therapies against neurodegenerative diseases [1]. If PEDF expression is decreased, spinal cord neurons are susceptible to nerve damage. This could lead to vertebral degeneration due to diminished bone mineralization capabilities [1]. Therefore, the loss of PEDF could disrupt the homeostasis of the CNS and manifest into the pathogenesis of spondylomyelopathy. Additionally, PGH2 is downregulated, with PGH2 increasing in treated groups of canines with spondylomyelopathy [1]. Typically, PGH2 is in abundance in the CSF; however, breakage in the blood-CSF barrier due to spinal canal narrowing could lead to a decrease in PGH2 concentration.

Apolipoprotein E and clusterin

Apolipoprotein E (APOE) is a key protein synthesized in the liver and is involved in lipid and fat metabolism [33]. APOE helps form lipoproteins; it is a key protein involved in cholesterol packaging and transporting cholesterol, fat-soluble vitamins, and lipids throughout the bloodstream [34]. In the immune system, APOE is involved in T-cell suppression, macrophage regulation, and the presentation of lipid antigens to natural killer cells [35]. APOE isoforms are also found to have neuronal repair mechanisms and neurotrophic properties [36].

Clusterin is encoded by the CLU gene on chromosome 8 and is a heterodimeric protein with many important cellular roles. Primarily, it functions as a chaperone for apoptosis along with clearing misfolded proteins, making it a neuroprotector [37]. Its ability to modulate apoptosis and arrest the cell cycle has allowed it to activate cell cycle control mechanisms, such as the phosphatidylinositol 3-kinase-protein kinase B (PI3K-AKT) pathway. Clusterin has also been described to be involved in inflammation regulation, lipid transport, cell differentiation [38]. It is also highly conserved in nervous tissue and is a major regulator of neurologic disease, including myelopathies [39].

**Figure 2:**
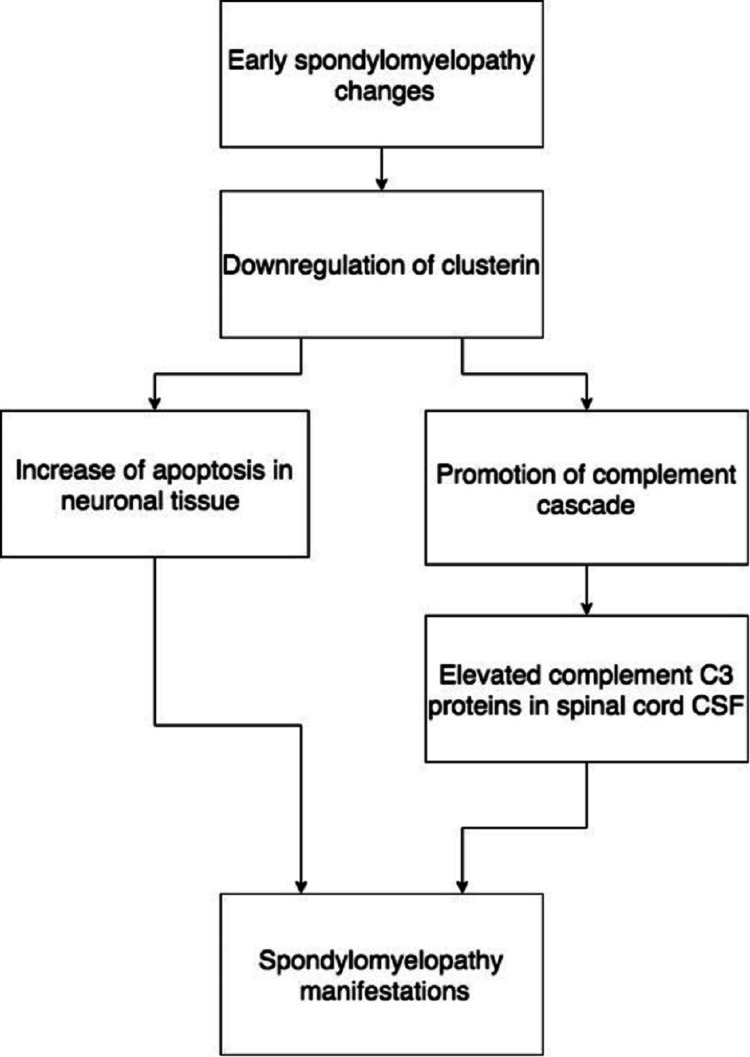
Mechanism of how spondylomyelopathy can lead to an increase in complement C3 proteins by decreasing clusterin protein expression. CSF: cerebrospinal fluid

APOE and clusterin are downregulated in degenerative myelopathies, with results from Martin-Vaquero et al. indicating that protein expression increased following treatment with corticosteroids [1]. Downregulated APOE will lead to a loss of neurologic repair mechanisms and is associated with worse surgical outcomes in patients with myelopathies [1]. Downregulated clusterin leads to a loss of apoptotic regulation, as apoptosis of neuronal and oligodendrocyte cells are involved in the pathogenesis of spondylomyelopathies [1]. In addition to the promotion of apoptosis, the complement cascade is upregulated without clusterin regulation, leading to neuronal tissue breakdown [1]. This relationship is linked with the complement C3 protein, as low clusterin leads to elevated C3 in the CSF of patients with the condition (Figure 2).

## Conclusions

The identified proteins are biomarkers of spondylomyelopathy and potentially other neurodegenerative diseases within the spinal cord. In addition, these proteins could be valuable targets for therapeutic interventions by either inhibiting or promoting their expression. However, despite our review, the proteomic profile of spondylomyelopathies remains poorly understood. There are limitations with regards to the number of spondylomyelopathy studies analyzing CSF in humans, along with the studies involving multiple treatment groups. Therefore, future studies are warranted to better understand which proteins are involved in the pathogenesis of spondylomyelopathy in humans, along with what risk factors could put a patient at risk of developing the pathology. These studies should also translate how certain proteins can contribute to specific degenerative and pain-related manifestations. Nevertheless, the analysis of proteins is significant to uncovering the pathophysiological uncertainties. In doing so, we can expect the enhanced characterization of disease progression, the establishment of disease biomarkers, and the possibilities for novel treatment options to improve patient outcomes.
